# Zoonoses in pet birds: review and perspectives

**DOI:** 10.1186/1297-9716-44-36

**Published:** 2013-05-20

**Authors:** Geraldine Boseret, Bertrand Losson, Jacques G Mainil, Etienne Thiry, Claude Saegerman

**Affiliations:** 1 Epidemiology and Risk Analysis Applied to Veterinary Sciences (UREAR-ULg), Department of Infectious and Parasitic Diseases, Faculty of Veterinary Medicine, University of Liège, Liège, 4000, Belgium; 2 Parasitology, Department of Infectious and Parasitic Diseases, Faculty of Veterinary Medicine, University of Liège, Liège, 4000, Belgium; 3 Bacteriology and Pathology of Bacterial Diseases, Department of Infectious and Parasitic Diseases, Faculty of Veterinary Medicine, University of Liège, Liège, 4000, Belgium; 4 Veterinary Virology and Animal Viral Diseases, Department of Infectious and Parasitic Diseases, Faculty of Veterinary Medicine, University of Liège, Liège, 4000, Belgium

## Abstract

Pet birds are a not-so-well known veterinarian’s clientship fraction. Bought individually or in couples, as families often do (which is a lucrative business for pet shops or local breeders) or traded (sometimes illegally) for their very high genetic or exotic value, these birds, commonly canaries, parakeets or parrots, are regularly sold at high prices. These animals, however, are potential carriers and/or transmitters of zoonotic diseases. Some of them could have an important impact on human health, like chlamydophilosis, salmonellosis or even highly pathogenic avian influenza A H5N1. This review paper, although non exhaustive, aims at enlightening, by the description of several cases of bird-human transmission, the risks encountered by bird owners, including children. Public health consequences will be discussed and emphasis will be made on some vector-borne diseases, known to be emergent or which are underestimated, like those transmitted by the red mite *Dermanyssus gallinae*. Finally, biosecurity and hygiene, as well as prevention guidelines will be developed and perspectives proposed.

## Tables of contents

1. Introduction

2. Main transmission routes

2.2 Vector borne transmission

2.2.1 Mites

2.2.1 Mosquitoes

2.2.1 Ticks

2.2 Direct contact

2.2.1 Households

2.2.1 Pet shops, bird fairs and markets

2.2.1 International trade

3. Most important diseases

3.1 Bacterial diseases

3.1.1 Chlamydophilosis

3.1.1 Salmonellosis

3.1.1 Tuberculosis

3.1.1 Campylobacteriosis

3.1.1 Lyme disease

3.1.1 Others

3.1 Viral diseases

3.1.1 Avian influenza

3.1.1 Arboviruses

3.1.1 Others

3.1 Parasitic/fungal diseases

3.1.1 Toxoplasmosis

3.1.1 Cryptococcosis

3.1.1 Others

4. Guidelines to prevent transmission from birds to humans

4.1 Household hygiene

4.1 Bird origin traceability

4.1 Awareness of sickness signs

4.1 Biosecurity and hygiene precautions in big facilities

5. Conclusions

6. Competing interests

7. Authors’ contributions

8. Authors’ information

9. Acknowledgements

10. References

## 1. Introduction

The term “Pet bird” designates birds housed and bred for an exclusively ornamental use. This category includes and will refer later in this paper to mainly passeriformes (e.g. canaries, finches, sparrows: see Table 1), also called songbirds, and psittaciformes (parrots, parakeets, budgerigars, love birds: see Table 1) [1-3]. It is a rather unknown vet’s clientship fraction. A statistical study made by the American Veterinary Medicine Association (AVMA) recorded 11 to 16 million companion and exotic birds in the United States in 2007 [4]. In 2010, following a study made by the FACCO (*chambre syndicale des Fabricants d'Aliments préparés pour Chiens, Chats, Oiseaux et autres animaux familiers*), 6 million pet birds are owned by French people [5]. In Belgium, every bred bird has to be identified by a ring sharing a number directly connected to the breeder’s owner (Arrêté du Gouvernement wallon fixant des dérogations aux mesures de protection des oiseaux, AM 2003-11-27). In 2011, the Association Ornithologique de Belgique (AOB) registered 249 ornithological societies authorized to identify their birds by an official ring.

**Table 1:** Main pet bird species according to the International Ornithologic Congress (IOC) classification 3.1 (2012)

**Order**	**Family**	**Genus**	**Species**	**English name**	**French name**
**Passeriforms**	Fringillidae	*Serinus*	*S. canaria*	Canary	Canari/serin des canaries
		*Carduelis*	*C. carduelis*	Gold finch	Chardonneret
			*C. chloris*	Green finch	Verdier
			*C. spinus*	siskin	Tarin
		*Pyrrhula*	*P. pyrrhula*	Bullfinch	Bouvreuil
		*Fringilla*	*F. coelebs*	Chaffinch	Pinson des arbres
	Estrildidae	*Taeniopygia*	*T. guttata*	Zebra finch	Moineau mandarin
		*Poephila*	*P. acuticauda*	Long-tailed finch	Diamant à longue queue
		*Erythrura*	*E. gouldiae*	Gouldian Finch	Diamant de Gould
		*Lonchura*	*L. striata*	Bengalese finch	Bengali/moineau du japon
	Sturnidae	*Gracula*	*G. religiosa*	Mynah	Mainate
		*Sturnus*	*S. vulgaris*	Starling	Etourneau
**Psittaciforms**	Psittacidae	*Melopsittacus*	*M. undulatus*	Budgerigar	Perruche ondulée
		*Agapornis*	*A spp*	Lovebird	Inséparable
		*Psittacula*	*P. eupatria*	Alexandrine parakeet	Perruche alexandrine
		*Lorius*	*L. spp*	Lories	Loris
		*Psittacus*	*P. erithacus*	African or Timneh grey parrot	Gris du Gabon
		*Poicephalus*	*P.senegalus*	Senegal parrot	Perroquet Youyou
		*Ara*	*A spp*	Macaw	Ara
		*Aratinga*	*A spp*	Conure	Conure
		*Amazona*	*A. aestiva*	Amazon	Amazone
	Cacatuidae	*Cacatua*	*C. alba*	Cockatoo	Cacatoës
		*Nymphicus*	*N. hollandicus*	Cockatiel	Calopsitte

Many families own their “kitchen pet bird”, which represents a lucrative business for pet shops or local breeders, since a single male canary is sold around 30 euros in Belgium and a female around 20 euros. Prices are about the same for zebra finches or budgerigars, and 50% to 100% higher for “special” finches like Gould diamonds. Bird fairs and live bird markets also gather a lot of people. In addition, some species are bred for their very high value; for example, in the case of canaries, male and female breeding stock reproducers with recognized genetic potential are presented in national and international contests for their posture (the Bossu Belge), their color (red mosaic) or for their song (Harzer). As a consequence, their offspring could be sold at high for rising prices. Finally, exotic birds like greater psittaciforms (parrots, e.g. ara or cockatoo), legally or illegally traded from for example Asia or South America, remain high in the ranking of popular pets and are also profusely represented in zoos and parks.

Notwithstanding these socio-economic facts, these animals are potential carriers and/or transmitters of zoonotic diseases. Some of these pathologies could have an important impact on human health, like chlamydophilosis, salmonellosis or even highly pathogenic avian influenza A H5N1, but also have an economic impact if some of these pathogens are spread via carriers or vectors like wild birds, human beings, insects or mites to poultry breeding units or cattle facilities [6], then entering the food chain. The aim of this review is to enlighten and discuss the risks encountered by bird handlers (including children), professional workers (e.g. veterinarians, traders or shop owners) in particular and the human population in general, and to assess the eventual health and economic consequences, and propose some guidelines to prevent transmission from such birds to humans.

## 2. Main transmission routes

Note: main transmission routes of diseases are summarized in Table 2.

**Table 2:** Main transmission routes of diseases

**Transmission route**	**Contagious diseases**				**Non contagious diseases**	
**Direct contact**	yes	yes	no	no	no	no
**Indirect contact**	yes	yes	yes	yes	no	no
**Vector-borne**	yes	no	yes	no	yes	no
**Example in pet birds**	Chlamydophilosis	Tuberculosis	West Nile Fever	Cryptosporidiosis	Lyme disease	Genetic disorders

### 2.1 Direct contact

#### 2.1. 1 Households

Passeriforms and psittacines are housed under different conditions, due to their respective behavior. Indeed, psittacines, especially parrots, are more aggressive than passerines and would then rather be kept in pairs than groups [2,3]. However, relatively high numbers of budgerigars can be gathered temporarily in the same cage for example in pet shop facilities or markets.

Besides the “kitchen-housing”, which is usually a single cage typically containing a couple of canaries or budgerigars for example, passeriform species are rather kept in captivity in two different types of aviaries [2]: mixed ornamental aviaries and breeding facilities. The first type is usually a big wire-netting space (up to 10 m^3^) located outside and sometimes with different species kept together, mostly for ornamental purposes [2]. In the second type, relatively large numbers of the same species, depending on the breeding size and breeding purpose (pet shops versus competitions) are maintained in pairs, mostly indoors (but sometimes with a partial access to the outside). In both types, new individuals are regularly introduced, in the first case with a purpose of ornamental diversification and in the second, to bring new blood into the genetic diversity of birds. These movements are supposed to be preceded by a quarantine of the new incomers.

Several times a year, performing birds are brought to shows and competitions, where exchange or selling could occur, and by the same way, transmission of pathogens, as this has been well illustrated by several authors [7,8]. In the case of the “kitchen-canary”, it could be interesting to mention that in the summer, the cages can be moved outside, in order to allow the bird to sunbathe. This could be a condition favoring contact between wild and captive passerines (Boseret, personal observations). It is also not rare for canaries to escape from their cage, with a potential risk of disseminating pathogens into a wild avian population, pathogens which they could have contracted in their original breeding facility or from humans (for example, chlamydophilosis [8]). Predators, like cats, could also be infected. The question whether bird predators could eventually become sentinels has to be raised and needs to be further investigated. Finally, one should not forget other potential zoonotic pathogens shedders, like arthropods or rodents, which could also find an easily reachable source of food in cages (Boseret, personal observations) or directly on birds themselves, as this could be the case for haematophagous insects [9,10].

#### 2.1.2 Pet shops, bird fairs and markets

In direct relationship with local breeders, housing of birds in pet shop facilities enhances the risk of transfer of several zoonoses, like for example chlamydophilosis [8]. Cages are indeed often overcrowded, filled with birds of mixed origin [8]. The overcrowding also induces intense stress to the birds due to fighting for females, territory (which is extremely limited in this case) or food. This will cause quick debilitation of the weakest individuals and higher sensitivity to infections [11]. This situation is particularly true in live animal markets as represented in numerous studies performed in Asian countries [12,13]. Unfortunately, no data are available for European countries. But it is frequently observed that pet birds share the same space as poultry, making transmission of pathogens and parasites easier (e.g. *Dermanyssus gallinae*).

Finally, bird fairs are a final example of possible contamination. In these regional, national or international gatherings, breeders meet each other and present their production, in a context of championships. Cases of transmission of *Chlamydophila psittaci* from birds-to-humans in France and The Netherlands in such conditions have recently been related by respectively Belchior et al. and Berk et al. [7,14]. In both cases, clinical symptoms were developed by patients and led in several cases to hospitalization.

#### 2.1.3 International trade

As illustrated by several authors, controlled as well as non-controlled movements of birds could enhance the introduction of zoonotic pathogens (like chlamydophilosis or highly pathogenic avian influenza A) and their vectors (like *D. gallinae*) in non-endemic countries [15-18]. Indeed it is still difficult to obtain an accurate estimation of wildlife trade since most of the time it is conducted through non-official and non-legal routes [19-21]. It must be pointed out that illegal wildlife trade for e.g. companion or ornamental pets, ranks second in terms of economic activities as compared to illegal narcotic trade [22]. In addition to this huge financial impact, this situation also reflects a non-negligible threat for human health since it facilitates multiplication and circulation of zoonotic pathogens and could facilitate adaptation of these pathogens to new hosts [16,22]. However, controlling movement is not the absolute way to prevent pathogen transmission. Roy and Burnonfosse have illustrated this fact through their study on nuclear and sequence data analysis of pest species [18] wherein the authors showed that commercial exchanges could have an impact on international gene flow in populations of *D. gallinae*, even in a highly controlled context (for example, quarantine measures in industrial layer farms).

### 2.2 Vector borne transmission

#### 2.2.1 Mites

Vector-borne diseases represent a major problem for public health. Bird ectoparasites, especially mesostigmatic mites belonging to Dermanyssidae and Macronyssidae, are well known for their heavy potential to transmit diseases to poultry. *Dermanyssus gallinae* in particular, even if exhaustively described in poultry breeding, is also an underestimated pet bird pathogen. This mite is often found in both the pet bird family household and intensive breeding. *D. gallinae* is a nocturnal haematophagous ectoparasite and has been described to cause an important debilitation by exsanguination, involving high mortality rate in newborns, and sometimes in hens. *D. gallinae* has also been proven to transmit zoonotic pathogens [23-25], such as *C. psittaci*[26], *Coxiella burnetii*[24,25], *Salmonella spp.*[27-29], *Erysipelothrix rhusiopathiae*[30,31], *Listeria monocytogenes*[24,25] and viruses like Fowl pox virus [24]. Moreover, evidence of transmission to humans has been described, with subsequent apparition of skin lesions and a dermatological pruritic syndrome. [32-38]*D. gallinae* is characterized by a specific thigmotactic behavior and spends most of its life in the bird’s environment rather than on the host itself, especially in narrow interstices like perches, feeding bowls and sand tray anfractuosities; it acts more like a mosquito or a bed bug than like other parasites, since it only occasionally bites its hosts to take a blood meal [39]. In addition to complicating early detection of the mite (on the contrary to other parasites spending most of their life on the bird, like e.g. the blood-sucking mite *Ornithonyssus silviarum*-see also below), this particular life trait makes the parasite hard to eliminate by antiparasitic spray treatment (e.g. organo-phosphorus, pyrethrinoids) [40]. A topic treatment, with application of a long-term remanent antiparasitic spot-on product (e.g. 0.1% ivermectine) directly on the birds’ skin, has been suggested by Dorrestein [41]; this alternative may not, however, be easily applicable in large breeding and big facilities.

*D. gallinae* could be considered as an invasive species presenting a wide host spectrum, of more than 40 bird families (including passeriforms [39,40]). The hypothesis has been formulated that these parasites could be easily transmitted horizontally, from one infested bird nest to another close one [42] or in the case of mixed colonies [43]) or from wild birds (e.g. passerines) feeding in open air together with domestic species [40]. This could also represent a way of transmission to humans. Indeed it has been shown that pigeons do nest in the vicinity of humans (such as city buildings, including hospitals [44]) and several case studies have presented the evidence of *D. gallinae* populations close to abandoned pigeons perches or nests, near windows or aeration circuitry [2,35]. Such infestations are in direct relationship with dermatologic clinical syndromes in humans (“pseudo-scabies”), associated with pruritic syndrome [32-38]. Since *D. gallinae* has been provento shed zoonotic pathogens [28,37] and since birds like pigeons have been found to be perching alongside hospital walls [35], one could point out the eventual risks encountered by immunocompromised humans, like hospitalized people, if they experience such a situation.

*Ornithonyssus sylviarum* (Macronissidae), also named Northern Fowl mite or white poultry mite is another blood-sucking arthropod identified in pet birds. Clinical symptoms are similar to those developed by a *D. gallinae* infestation: depression, anemia, newborn mortality [41]. However, *O. sylviarum* behavior is notably different from *D. gallinaes*, since it spent its entire life on the host’s body, making pest detection in some way easier [41,45]. *O. sylviarum* has been isolated in wild avifauna and pet birds; it has the ability to leave its host and reach birds even housed in other cages. However, its capacity to resist from starvation (i.e. living in the absence of any host) in the environment is significantly shorter than the red mite’s (resp. 3 weeks and 24 weeks [45]). Only a few cases of zoonotic transmission to humans have been reported, with clinical signs restricted to dermatologic symptoms associated with pruritus [46]. Nonetheless, *O. sylviarum* is considered to be emergent in Europe and to present an increasing problem in aviaries [41] and should therefore not be neglected.

#### 2.2.2 Mosquitoes

Different species of mosquitoes (*Diptera*, especially *Culex* species) are responsible for horizontal and reciprocal transmission of arboviruses like West Nile fever Virus (WNV; [47,48]) or Usutu virus [49]. These diseases will be discussed further in the next section.

Dipterae act as bridging vectors between two host categories: amplificators (e.g. birds) and incidental/dead-end (e.a. humans). According to Turell et al., Sardelis et al. [50,51], cited in [47], an infected vertebrate must present a viremia of 10^5^ pfu/mL (pfu: plate forming unit) to be efficient as an amplification host. Studies have shown that house sparrows develop WNV viremia superior to 10^10^ pfu/mL after experimental infection, and maintain it above 10^5^ pfu/mL for five days [47,52,53] and they are indeed good amplificatory hosts and, moreover overwintering hosts [48] for at least one arbovirus, the WNV. Beside these effects of amplification and seasonal resistance, international exchanges, trade and migration are factors enhancing the emergence of these viral diseases, as shown by the increasing number of diagnosed infections acquired during stays in tropical countries. Interestingly, Pfeffer and Dobler [53] pointed out the fact that no attention is actually paid on accompanying pet animals and the parasites that these pets could be carrying. Pet birds are also important since a large number of companion birds are obtained by sellers from trade with exotic countries [15,53].

#### 2.2.3 Ticks

Ticks from the genus *Ixodes* (e.g. *I. ricinus*, *I. scapularis*), are carried by birds and then have the ability to transmit pathogens like *Borrelia burgdoferi*, the causative agent of Lyme disease, and the flavivirus louping ill virus. Migrating birds could also be carriers of infected ticks and then contribute to long distance dispersal of both vectors and spirochetes [54]. Mathers et al. recently published an interesting study on the potential role of wild birds and the ticks that feed on them in the introduction of the Lyme disease agent to emergent areas [55]. No evidence, however, has been reported on transmission from wild to domestic pet birds even housed in open air aviaries.

## 3. Most important diseases

Note: Tables 3 and 4 summarize the main diseases described below in terms of clinical signs and gross lesions presented by birds, recommended diagnostic tools and treatment, and symptomatology reported in humans.

**Table 3:** Summary of main pet bird zoonotic diseases

**Disease**	**Pathology**	**Clinical issue**	**Asymptomatic shedding**	**Transmission route**	**OIE listed disease**	Risk for humans*
**Chlamydophilosis**	Systemic	Fatal	yes	D/I/V	Yes	high
**Salmonellosis**	Digestive to systemic	Treatable	yes	D/I/V	No	moderate
**Tuberculosis**	Respiratory to systemic	Fatal	no	D/I/V	Yes	high
**Campylobacteriosis**	Digestive to systemic	Treatable	yes	D/I/V	No	moderate
**Lyme disease**		None	no	V	No	low
**Avian Influenza**	Systemic	Fatal	no	D/V?	Yes	high
**West Nile fever and other arboviruses**	Respiratory to systemic	Fatal	yes	V	Yes (WNF)	moderate
**Avian Bornavirus**	Digestive/nervous to systemic	Fatal	no	D	No	null
**Newcastle disease**	Ocular To Systemic	Mild to fatal	yes	D/I/V	Yes	low
**Toxoplasmosis**	Digestive	Digestive	yes	I	No	Null to low
**Giardiosis *(G. duodenalis)***	Digestive to systemic	Treatable	yes	I	No	moderate
**Cryptosporidiosis**	Digestive	Treatable	yes	I	No	moderate
**Cryptococcosis**	Digestive	Treatable	yes	I	No	moderate

*when handling a bird without hygienic precautions.

Legend: D = direct contact; I = Indirect contact; V = vector-mediated contact.

**Table 4:** **Summary of clinical data associated with main pet bird zoonotic diseases [**[41]**]**

**Disease**	**Sensitive species**	**Clinical signs**	**Necroptic lesions**	**Diagnostic (sample/analysis)**	**Remarks and Pitfalls**	**Treatment**	**Human symptoms**
**Blood-sucking mites**	All	*Nestlings*: weakness, anemia, death *Adults*: AA, respiratory distress, depression	None	Direct examination	*Dermanyssus gallinae*: hide in cages anfractuosities and could not be found on birds themselves	Ivermectine, permethrins in spray. Total disinfection of cages and facilities (see also chapter 4)	Dermatitis, pruritus
**Chlamydo-philosis**	Psittacines – canaries – finches	AA, SBS, diarrhea, nasal discharge, dehydration, Ocular signs	Air sac lesions, hepato-splenomegaly	CSw, OSw, FE/BC, serology (paired serology 2 weeks apart),IMF, PCR	Asymptomatic carriage (up to 40%), false negative	Tetracyclins (1^st^ of 2d generation)	Flu-like syndrome, genital, articular, skin symptoms
**Salmonellosis**	All (open-air aviaries)	AA, WL, diarrhea, mild respiratory symptoms	Congestive gastro-intestinal tract, hepato-splenomegaly	CSw, FE	Mostly in winter and in outdoor aviaries; hard to differentiate from pseudo-tuberculosis	Not recommended (high probability of antibio-resistance)	Gastro-intestinal infection
**Tuberculosis**	Psittacines (canaries?)	Progressive AA, WL, respiratory symptoms, long bone lesions	Cachexia, osteolysis spots in long bones, lung lesions (non caseous)	RX (bone lesions), OSw/MO (Ziehl-Nielsen), BC, HP	Chronic development, sometimes during months to years; human origin infection	Not recommended (high probability of antibio-resistance)	Chronic pulmonary symptoms (caseous lung knots), generalized infection
**Campylo-bacteriosis**	Estrildidae mostly.	Apathy, yellow feces (solid or liquid)	Cachexia, congestive gastro-intestinal tract, containing a yellow amylum or undigested seeds.	FE/MO (curved rods in stained smears), BC	Canaries and psittacines are asymptomatic carriers	Not recommended (high probability of antibio-resistance)	Gastro-intestinal infection, Gillain-Barré syndrome
**Avian Influenza**	Passerines	Sudden death, SBS, respiratory and neurological signs	Dehydration, respiratory lesions	OSw, CSw, BS/HP, PCR	Mostly in outdoor aviaries	None	Mild to severe respiratory and systemic infection
**West Nile fever**	All	Ocular and neurological signs		OSw, CSw/PCR	Mostly asymptomatic carriage	None: prevention based on limitation of exposure to mosquitoes (vectors)	Mild to severe respiratory and systemic infection, encephalitis, septicaemia, death
**Newcastle disease**	All	SBS, AA, ocular, respiratory and neurological signs	Dehydration, respiratory lesions	OSw, CSw/serology		None; prevention by vaccination	Cunjunctivitis, mild flu-like symptoms
**Toxoplasmosis**	Canary, finch, budgerigar minah	SBS, AA, respiratory and neurological signs, blindness	iridocyclitis, panophthalmia, catarrhal pneumonia, hepato-splenomegaly	CSw/MO, serology, HP, PCR	Systemic symptoms sometimes unseen; detection of the disease 3 months later (blindness)	Trimetoprim-sulfamids	Mostly asymptomatic. Abortion, congenital malformation.
**Giardiosis (G. duodenalis)**		None	None				Sometimes asymptomatic. WL, diarrhoea, abdominal pain
**Crypto-sporidiosis**	All	Rare; acute diarrhoea	Gastro-enteric lesions	CsW/MO		Ronidazole	Gastro-intestinal symptoms; liver, pancreas, respiratory tract lesions
**Cryptococcosis**	Parrots, little pet birds	Rare	None	CSw/MO	Possible aerosol-borne contamination		Mostly asymptomatic. Respiratory and nervous symptoms.

*AA* apathy-Anorexia, *WL* weight loss, *FE* feces examination, *BC* bacterial culture, *MO* microscopic observation, *SBS* sick bird signs (ruffled feathers, standing at the bottom of the cage, depression), *HP* histopathology (including immunocytochemistry), *BS* blood sample, *CSw* cloacal swab, *OSw* oral swab, *IMF* Immunofluorescence, *PCR* polymerase chain reaction.

### 3.1 Bacterial diseases

#### 3.1.1 Chlamydophilosis

One of the most threatening zoonotic diseases transmitted by birds to humans is chlamydophilosis (also known as chlamydiosis, ornithosis, psittacosis or parrot fever), caused by the intracellular bacterium *Chlamydophila psittaci*. Psittacine species are highly sensitive to this pathogen, but passerines are not excluded [26,41,56]. Human symptoms come from mild respiratory signs to severe pneumonia, with localization in several organs leading to diarrhea, conjunctivitis, arthritis and genital organ infection. The first people susceptible to be infected appear to be, as expected, veterinarians and birds breeders; this has been e.g. enlightened by the two following studies. The first reported an accidental contamination of a vet by infected turkeys [57]; the second, an epidemiological study made by Ghent university, pointed out a high percentage of human infection in owners and vets working in breeding psittacine facilities [8]. On 39 breeding facilities, which represent 308 birds (most of them are psittacines (e.g.): cockatoos, parrots, parakeets or lories) and 46 humans, 19.2% of birds tested positive for *C. psittaci* by nested PCR/EIA, 13% of pet owners (and the vet student in charge of the study) also tested positive after swap pharyngeal sampling. Sixty-six percent of the positive people presented mild respiratory symptoms, in association with viable *C. psittaci* isolation. Van Rompay et al. concluded their investigation with an important observation: on 18 breeding facilities, despite a broad spectrum-antibiotherapy, 60.6% were still positive for *C. psittaci* through culture and PCR (16.6% and 44% respectively) [8]. This raises the point of antibiotic resistance and development of drug-resistant strains in some facilities.

Another interesting case was described in a Liège hospital (Belgium), where a 10-year old child was admitted for persistent fever, acute abdomen, pneumonia and neurologic symptoms [58]. The pathogen, further identified as *C. psittaci*, was cefotaxime-resistant. Two budgerigars (the second most popular pet bird) were housed in the child’s living place; the elder brother of this child presented a high level of anti-*C. psittaci* IgA, which suggested a non-symptomatic chlamydophilosis.

Direct transmission of *C. psittaci* from birds to humans is underlined in a compendium of security measures about avian chlamydophilosis edited by the Centre of Diseases Control and Prevention in 1998, warning bird owners (43% of infected people in the USA between 1987 and 1996) but also professionals working with birds like e.g. veterinarians, breeders, zoo workers… to be aware of a real risk of zoonotic transmission [59]. Bird fairs are a good illustration of the occupational risk presented by a high concentration of people and birds in the same space for a relatively long period of time. Belchior et al. and Berk et al. reported recently two similar events in respectively France and The Netherlands, where chlamydophilosis outbreaks occurred during bird fairs. In Belchior’s study, 68% of the exhibitors tested positive for *C. psittaci* infection [7,14].

Finally, one has to mention a case of illegally imported *C. psittaci*-positive psittacine occurring at the Antwerp custom office, that led to the hospitalization of custom officers after handling infected parakeets [15,17].

This points out the real threat pet birds could represent when little information on biosecurity is provided to the people breeding and/or handling them. *D. gallinae* could moreover transfer this pathogen [24-26]. This reinforces the urgent need for hygienic measures to be applied in places of risk, i.e. at bird fairs, pet shop facilities and small familial breeding units. The CDC compendium of measures to control *C.psittaci* would be of great help [59].

#### 3.1.2 Salmonellosis

*Salmonella* species were isolated from several captive passerine or psittacine birds, in relation or not (asymptomatic carriage) to clinical symptoms: diarrhea, multisystem disease, septicaemia, osteomyelitis, depression, crop stasis, dehydration, anorexia [56,60-63]. The serovar Typhimurium, a well-known zoonotic agent, was described in passerine birds in such clinical manifestations as granulomas (liver, ceca, spleen), multisystemic symptoms, ocular lesions and osteomyelitis [61,64]. Transmission to humans was reported in different cases [63,65,66]. Smith et al. also reported two cases of *Salmonella typhimurium* outbreaks in elementary schools related to owl pellet dissection [67]. Even if these cases are more anecdotal than quite frequent, people should be careful (and at least respect elementary hygienic rules) when manipulating bird products such as wild bird pellets, which could be in a somehow comprehensive way undertaken as a didactical manner to teach nature to kids. Another point of view is the problematic of wild reservoirs. Indeed, wild songbirds have been repeatedly documented as *Salmonella spp.* carriers [68,69] and implicated in the transmission of these pathogens to humans and mammals. Starlings in particular, have been shown to be potential spread agents of salmonellosis in cattle feeding operations [6]. In addition, bovine herds have been demonstrated to be reservoirs of many gastro-intestinal pathogens being of concern to humans, especially professionals like livestock producers or veterinarians [70], as well as consumers [71].

Finally, as discussed in the chapters above, *D. gallinae* seems to play a significant role in *Salmonella spp*. Transmission in layer farms, as developed by Valiente Moro et al. [23,27-29].

#### 3.1.3 Tuberculosis

Isolation of zoonotic agents from the *Mycobacterium* species is not so rare in pet birds, especially in psittacines. The most commonly isolated species are respectively *Mycobacterium genavense* and *Mycobacterium avium*[56,72]. The main species causing tuberculosis in humans, i.e. *M. tuberculosis*, has only rarely been reported in birds, and essentially in parrots. In this particular bird family, an interesting observation should to be pointed out, especially since it seems that the main route of infection was of human origin. Well documented examples are the green-winged macaws (*Ara chloroptera*) diagnosed positive for *Mycobacterium tuberculosis*, the first in New York City [73] and the second in Switzerland [74]. Both birds developed a panel of clinical signs associated with tuberculosis: lethargy, osteomyelitis, multifocal granulomatous panniculitis and granulomatous hepatitis. In both cases, the bird owners had a history of culture-confirmed pulmonary tuberculosis and confessed real close contact with their birds (mouth-to-beak feeding). Moreover, in the Swiss case, two veterinarians in charge of the case showed a positive reaction to tuberculin skin test after handling the sick bird [74]. One observation made by the authors is that these parrots have lived a sufficiently long time while incubating the disease to become themselves a potential source of infection for other humans. Data are lacking on the susceptibility of non psittacine pet birds to *M. tuberculosis*, since the authors found only one study reporting such an infection in a canary, diagnosed with a lung knot positive for *M. tuberculosis*[75].

It is, however, a fact that infection with zoonotic *Mycobacterium spp* in pet birds is rare. Regarding the susceptibility of birds to *Mycobacterium bovis*, to date, only experimental infections have been reported as being responsible for clinical signs. A recent study focusing on the experimental infection of budgerigars by several species of *Mycobacterium* reported that the only clinical signs were seen 70 days after inoculation with *M. bovis*, while no clinical signs were observed following the challenge with the other species [76]. *M. bovis* is also a zoonotic agent, considered to be responsible for 1 to 2% of human cases of tuberculosis in industrialized countries, while this proportion is susceptible to be much more important in developing countries (until 8% of human cases, depending on the region) [77,78].

Nevertheless, infected/carrying/untreated birds could become a potential reservoir for humans, and then have consequences on public health. In an ideal situation, surveillance and early diagnosis of zoonotic mycobacteria should be performed in every imported bird bunch [79,80] including animals captured from the wild [81]. Mycobacterial culture or PCR analyses would be the most sensitive and specific laboratory tests for a definitive diagnosis [82]. However, the long-term onset of the disease, the pathogen’s intracellular localization and the difficulty to dispose of not expensive highly sensitive diagnostic tests makes systematic and/or regular check-ups difficult to perform in routine conditions.

#### 3.1.4 Campylobacteriosis

*Campylobacter spp.,* and in particular *Campylobacter jejuni* are responsible for food-borne diseases in many countries, responsible in humans for debilitating symptoms such as gastro-enteritis (diarrhea, vomiting), headaches, and depression, leading sometimes to death. Campylobacteriosis was the most frequent zoonotic disease reported in 2009 in the European Union [71]. But *Campylobacter spp.* is not exclusively a food-borne disease. Even if little information is available on the role of other avian species (like pet birds) in the epidemiology of the disease, this pathogen is shed by an important bird variety, among which are “hobby birds” including estrildidae, canaries and psittacines [41,83,84]. Moreover, an Italian study showed a high occurrence of *C. jejuni* in migrating passeriforms [85], and concluded that these birds constitute a reservoir and a possible transmission route from birds to humans and domesticated animals, including cattle. This observation was also made by Adhikari et al. in 2004 [86], in a study dealing with dairy cows and sparrow fecal matters in New Zealand. However, other reports and experimental protocols tend to demonstrate that *C. jejuni* infection is highly host-specific and that transmission from birds to humans, *a fortiori* from pet birds, although not impossible, is likely to play a minor role [87,88]. Nevertheless, one still has to consider the potential role of pet birds in *C. jejuni* shedding and consequently apply elementary hygienic precautions while manipulating birds and/or feces.

#### 3.1.5 Lyme disease

Different strains of *Borrelia burgdorferi sensu lato* were isolated from ticks collected on songbirds in different areas of the world, including Europe [54]. Olsen et al. [89] showed that canaries presentrelatively quickly a mild spirochetemia after experimental infection with *B. burgdorferi,* but without or few clinical symptoms. This suggests that passerines may be of little importance as long-term amplifying reservoirs for borreliosis. Moreover, ticks are usually quickly detected in the feathers of bred birds, as well as in kitchen-canaries, diminishing then the risk of wild-to-captive bird transmission and a fortiori to humans.

Concerning psittacines, no evidence of Lyme disease seems to have been shown.

#### 3.1.6 Others

There are numerous other potential zoonotic bacteria also identified in pet birds, including multiple gram-negative bacteria such as *Pasteurella* spp, *Klebsiella* spp, *Yersinia* spp, *Pseudomonas spp.*, and *Escherichia coli*[41,56,90,91]. Indeed, *Escherichia coli* O157:H7 strains transmitted from wild passerines (European starlings mostly) to cattle and then introduced into the food chain has been reported in several studies [92-94]. Lack of hygiene and the absence of quarantine (especially concerning imported birds), and dirty food and water sources seem to be the most probable origin of infection with these zoonotic pathogens. Besides, the potential transmission from wild birds to open-air aviaries hosted pet birds (via fecal drops) should be considered (Boseret, personal observations). However, reports of transmission of these bacteria from pet birds to humans still lack in the literature.

### 3.2 Viral diseases

#### 3.2.1 Avian influenza

Highly pathogenic avian influenza A H5N1 has been in the world health focus since the year 2000’s outbreaks. Perkins and Swayne [95], demonstrated in 2003 that the avian influenza A virus H5N1 after intranasal administration was able to induce clinical symptoms leading to death in pet bird species like zebra finches and common budgerigars, which are very common hosts of domestic ornamental aviaries, as well as in wild species like house sparrows and European starlings, usually living close to human habitations [95]. Several studies demonstrated the important role of migrating birds as pathogen vehicles all over the world [21,96,97], being putatively able to infect wild indigenous birds (house sparrows, European starlings), these latter possibly contaminating pet birds living in open air aviaries [2]. This virus could also spread from endemic countries [12,16] to other locations through international trade of exotic birds [15,16,22]. In relation with this fact, markets where live birds are sold appear to represent a great risk for zoonotic transmission as demonstrated by several authors [12,13]. This is indeed noticeable that Asian owners seem to be, even at the peak of the H5N1 outbreak, unaware of the zoonotic risks this kind of business could cause [12,13] and this was also the case in Western countries as hybrids between canaries and different wild passerines were and are still sold on public markets (Boseret, personal information). Illegal bird importation can also induce a risk as suggested by Van Borm et al. [16].

#### 3.2.2 Arboviruses

West Nile Fever is an emergent vector-borne zoonosis in which birds, e.g. house sparrows, play a key role as main and amplifying reservoir hosts [48]. The virus responsible for this disease is a flavivirus (*Flaviviridae*) known under the name of the West Nile Fever Virus (WNV) which was isolated from numerous passeriform species, including canaries [48], as well as psittacines [98]. Most of the time Birds are sub-clinically affected, but can, however, develop a clinical form of the disease with ocular and neurologic symptoms [56]. Usutu virus (USUV) is another mosquito-borne flavivirus of African origin. This avian virus is transmitted by arthropod vectors (mainly mosquitoes of the *Culex pipiens* complex). Since 2001, the death of birds especially passerines, has been associated with infection by USUV [99,100]. It is well known that free-living birds, including migratory species, have the potential to disperse certain pathogenic microorganisms [53]. Usutu virus has recently been detected in Europe and is spreading through Austria, Hungary, Italy, Spain and Switzerland, causing disease in birds and humans [49]. Following the same pattern as the West Nile Fever virus, USUV is a candidate as an emerging pathogen in Europe and the consequences for human health safety have to be considered [49,53]. Open air aviaries are common in our countries and could be an important feeding source for mosquitoes, which could then inoculate the virus to humans.

#### 3.2.3 Others

Proventricular dilation disease (PDD) is a disease in pet birds and, since it could be frequently lethal, PDD is considered as a major threat to aviculture [101]. This syndrome is associated with inflammation of the nervous system and gastrointestinal dysfunction as well as neurologic changes like seizures. Recently, the cause of this disease has been attributed to a novel bornavirus, the Avian Borna Virus (ABV) [102]. However, there is no evidence of ABV cross-species transmission and the zoonotic potential of this family of viruses remains unclear [103].

Newcastle disease, caused by avian paramyxovirus (APMV) has also been described in pet birds [56,91,104]. Transmission to humans is also possible, with conjunctivitis [56] but the most important consequence would be spreading the infection among poultry breeding by the intermediary of humans, wild birds (especially pigeons) or maybe insect mechanical vectors like the house fly (*Musca domestica*) [105].

### 3.3 Parasitic/fungal diseases

#### 3.3.1 Toxoplasmosis

Toxoplasmosis is a well-known human disease, responsible for abortion or congenital malformations in humans. Although less documented than through the cat-cycle transmission, *Toxoplasma gondii* has also been described as an important pathogen for canaries, finches and budgerigars [106,107], inducing blindness among other symptoms. However, transmission to humans appears to be most unlikely, since the birds do not excrete *T. gondii* in feces (implying no risk of contamination by lack of hygiene or fecal matter manipulation). Indeed, *Toxoplasma gondii* should be found in internal organs and muscles, but since these birds are not usually bred for food purposes, this then eliminates the possibility of contamination by raw or undercooked flesh eating (Losson, personal communication).

#### 3.3.2 Cryptococcosis

Pigeons are known to be reservoirs of pathogenic yeasts, like *Cryptococcus neoformans*, which is described to cause opportunistic infections in humans [108]. However less is known on the role that pet birds might play in such zoonotic transmission. Several studies have demonstrated the presence of *C. neoformans* in parrots, little pet birds like canaries, budgerigars or lovebirds and cockatiels [109,110]. As discussed above, pet birds, housed in outdoor aviaries could be a potential health hazard for humans as *C. neoformans* reservoirs since they could be in contact with wild pigeon droppings.

#### 3.3.3 Others

Despite a relatively poor documentation on pet bird parasitic diseases, giardiosis, aspergillosis and cryptosporidiosis have been reported in these avian populations, both in chronic and in acute infections. Favorizing conditions could be high-density populations, stress, and adaptation to a new environment or prolonged periods in confined housing [111]. Transmission to humans often results from feces manipulation or lack of hygiene [41,56,90].

Avian giardiasis is caused by two different *Giardia* species: *G. ardeae* and *G. psittaci*. *G. psittaci* has been demonstrated to be responsible for fatal infections in budgerigars [112], but is not transmissible to humans. The species responsible for zoonotic infections is *G. duodenalis*, generally causing a self-limited illness, sometimes asymptomatic, characterized by diarrhoea, abdominal pain and weight loss [112]. *G. duodenalis* is divided into eight genotypes or “Assemblages”, among which the Assemblages A and B appear to be responsible for human infections [113]. Interestingly, these genotypes have been isolated in the feces of different avian species, without leading to the development of clinical symptoms. Birds then seem more likely to serve as mechanical vectors of cysts and oocysts [111].

In birds, *Cryptosporidium* infection leads to intestinal, respiratory or nephrotic symptoms and could be caused by three distinct species: *C. galli*, *C. meleagridis* and *C. baylei*. The latter two have been described as possible zoonotic agents, though in a low frequency in comparison with other species such as *C. hominis* or *C. parvum*[114]. The main human population at risk is very young children (first exposure, lack of hygiene) and immunocompromised individuels such as HIV-positive patients, who will develop gastro-intestinal lesions but also infections of other organs such as the pancreas, liver and occasionally the respiratory tract [115]. *C. parvum* has been isolated in feces of various avian species, corroborating the possibility of zoonotic parasite shedding and transmission by birds [116].

Aspergillosis has been frequently isolated from pet birds [56,117], in both acute (severe respiratory condition with lethargy and changes in vocalization) and chronic forms (more often fatal because of its long-term development). However, human infection would rather come from environmental origin, and therefore be considered as a minor zoonotic threat, apart from human immunocompromised patients [117].

## 4. Guidelines to prevent transmission from birds to humans

One interesting document to start with is the “Compendium of Measures To Control *Chlamydia psittaci* Infection Among Humans (Psittacosis) and Pet Birds (Avian Chlamydiosis), 1998” edited by the Centre for Disease Control in 1998 [59].

### 4.1 Household hygiene

The transmission of zoonotic pathogens from animals to humans could be easily decreased by applying some elementary hygiene principles. A few recommendations could be delivered to the owner by the bird seller:

•Clean clothing and shoes after any contact with other birds (bird club meeting, bird fair, live poultry).

•Wash hands before and after handling birds (including cage cleanings).

•Check cages, food and water every day to be sure they are clean (including perches, feeding cups, etc.…).

•When giving fruits or vegetables to birds, discard the rotten remainings.

•Change bath pots every day and make them available to birds only one hour/day (to avoid the bathing waste water to become a reservoir for pathogens).

•Wash cages once a week.

•Preserve food in clean and sealed containers.

•Clean and disinfect every aviary item before use.

Usually, bird breeders are correctly aware of these precautions; the risk is, however, higher in the case of family pets bought for the first time in a decorative purpose or as present for children, especially when either parents or children have not been informed about the cited above elementary advice.

### 4.2 Bird origin traceability

In the case of birds bred in the country where they are sold (e.g. little birds like canaries, finches, budgerigars), they are usually provided without any certificate or identification (apart from a leg band with the breeding identification number). Sellers are supposed to keep an accurate traceability of their stocks, but as far as we know there is no legal obligation for the seller to give any documents to the buyer.

For exotic pet birds issued from importation, laws differ from countries, but in a general view, a vet certificate, a passport and an importation authorization have to be delivered with the birds. As said before, smuggled birds represent a high risk of introducing zoonoses. In Europe, exotic bird importation from non EU countries is forbidden and animals imported from other EU-member countries should have an international passport, a correct identification and a veterinary certificate of good health (Directive 91/496/CE).

However from the owner’s point of view, there are some recommendations to be aware of after buying a new pet bird:

•If the bird comes from another country, request certification from the seller that these were legally imported (eventually ask for official documents) and were healthy prior to shipment (certified by an official veterinarian).

•Schedule an appointment with a veterinarian.

•Isolate new birds from other birds for a quarantine time determined by the veterinarian.

•Restrict access to birds from people owning birds too.

•Keep birds away from other birds (e.g. in the gardens).

### 4.3 Awareness of sickness signs

Breeders usually know signs of sickness in a bird, even if they could be somehow difficult to detect. But for non-initiated people, like sellers in animal shops or new owners, this could be difficult to see whether their birds are healthy or ill. Prevention tools and information should then be provided by the breeders to people to whom they are selling/giving their animals. Veterinarians should also better inform owners for example by providing documentation on warning signs of infectious bird diseases. If unusual signs of disease or if unexpected deaths occur in a breeding, the owners should then warn their avian veterinarian.

### 4.4 Biosecurity and hygiene precautions in big facilities

When of sufficient size, a Hazard Analysis and Critical Control Points (HACCP) plan could be applied in breeding facilities and in selling facilities. To quarantine newly incoming birds is an absolutely necessary precaution. These animals should be kept in clean cages for a duration estimated by the sanitary veterinarian, and pathogens and/or pest absence (including *D. gallinae*) should be carefully checked. CDC recommends at least a quarantine of 30–45 days when *C. psittaci* infection is suspected [59]. For example, one should check these different control points:

1. Direct bird environment:

Presence/absence of *D. gallinae* in the quarantine cages after at least one week, which is the time needed by the parasite to accomplish a complete reproduction cycle, from egg to egg [40]. For example, the acarids could be easily found on feed balls, perches or on the removable bottom sand tray. An easy test is to push strongly with the thumb on dirty spots pasted on the reverse face of this tray and scratch them from left to right (or vice versa). If a bloody smear does appear, this would be an efficient sign that blood-fed parasites did begin to colonize cage anfractuosities (Boseret, personal observations).

Color/consistency/quantity of droppings: for example, a yellow stain could suggest campylobacteriosis, a liquid consistency could refer to salmonellosis or other enterobacteriaceae infections [41].

Transport cages: were they soiled or clean? Presence of dead birds?

2. Birds: general examination

Presence/absence of other pest species living most of their time on the host, e.g. at the calamus of the feathers (like *O. sylviarum)*, at the edge of the beak or in the leg’s scales (like *Knemidokoptes pilae,* which is a non-zoonotic mange agent*)* or in another part of the body (e.g. ticks, lice). Broken feathers or feather-loss could indicate pruritus and discomfort, other indicators of ectoparasite infestation [41]. Ectoparasites are considered by many breeders to be a good indicator of inadequate hygiene and management and their detection therefore could awaken attention of the owner to the health status of their infested incoming birds.

General state of the birds (good/bad)

••Perching/lying at the bottom of the cage,

••Normal activity/apathy, rolled in ruffled feathers,

••In social groups/isolated,

••Bright eyes/exophthalmia,

••Good respiratory state/nasal-ocular discharge, open beak.

Plumage aspect: are the birds in a molting period? How is the molting: homogenous and bilateral/heterogenous and asymmetric.

3. Quarantine facilities hygienic state:

Frequency and efficiency of cages/walls/floor/shells disinfection,

Food storage (access to mice, rats?),

Environmental conditions: temperature, humidity, duration of light hours.

This list is not exhaustive and a complete list of adequate control points has to be determined as a function of the kind and size of breeding, facility conformation, season, frequence of bird movements, etc. The above recommendations should, however, constitute a basis of elementary examinations to be performed in every case.

In case of a high level of risk or when a doubt emerges relative to the birds’ health state, the following laboratory analyses could be performed:

1. Individual level: necropsy of a dead or a sacrificed sick bird, performed along with bacterial analyses of intestinal content or other organs presenting lesions.

2. Group level: Bacterial analyses of cloacal and/or oral swabs of a bird’s sample bunch (one-to-ten, one-to-fifteen…).

3. Vector level: molecular analyses of vectors found on birds and/or in the cages, to specifically detect zoonotic agents: *C. psittaci*, West Nile fever, etc.…

The first two types of analyses could be an interesting investment and should not be too expensive (less than 100 euros/bird’s bunch).

However, molecular analyses are on another financial level. One should recommend them in particular cases, first when birds are about to be handled by owners, like parrots, parakeets or cockatiels, second when the pathogen targeted is of zoonotic non negligible importance. For example, tuberculosis detection has to be carried out with a critical mind, as false negatives do occur. On the contrary, since surveillance of zoonoses is a European legal obligation (Directive 2003/99/EC), testing birds could be systematically included in national surveillance programs, a fortiori when human health is estimated to be put at risk. Then the breeders would be able to obtain an official intervention budget.

Another suggestion to diminish the costs at a local level would be to perform such tests in multiplex series, allowing breeders to somehow share elevated costs. But all these possibilities involves a complete change of mind and implies a broader transparency in these kinds of breeding, which still lacks even in our high-controlled countries (Boseret, personal information).

When birds are proven to be healthy, then they could be introduced in their definitive facilities. Outcoming birds should undergo similar sanitary systematic checking.

Moreover, the precaution of an all-in/all-out replacement system, already applied in poultry exploitations, should be carried out in pet bird breeding too. For example, only birds of the same age should be kept in the same location, and when moved, the facility should be disinfected carefully before welcoming a new flock.

In selling facilities, where birds from different origins could be mixed up, only replace them when the whole flock has been sold and the cages cleaned with ad hoc disinfectants. One interesting initiative would be to create a certificate of “good health” to moving flocks, but since many animals are sold in non-official ways (e.g. private breeding, markets), this would not be so easy to set up.

Control point should also be implemented on bird fairs. Sanitary certificates could be an obligatory document to be provided to authorities to allow the breeder to attend any fair.

## 5. Conclusions

This review aimed to present a non-exhaustive panorama of data relative to pet bird-human pathogen transmission. Different situations have been illustrated in this short review: familial households, breeding or selling facilities, bird fairs, international trade and the wild birds’ problematic of reservoirs. Although this represents a minor part of the companion animal vet clientship, pet bird diseases with zoonotic potential should not be neglected or underestimated, considering the major health impact on the population, including children. Referring to Pastoret and Vallat’s zoonoses classification, pet bird zoonoses are responsible for the most threatening disease types: 2 and 2+ (see Table 5; [118]). Vets could then play an important role in educating pets (including bird) owners.

**Table 5:** **Classification of emerging zoonoses [**[106]**]**

**Transmission**	**Wild to humans**	**Humans to humans**	**Wild to domestic**	**Domestic to humans**	**Example in pet birds**
**1**	Yes	No	No	No	None
**1+**	Yes	Yes	No	No	None
**2**	Yes	No	Yes	No	West Nile fever
					Newcastle disease
**2+**	Yes	Yes	Yes	Yes	Avian Influenza
					Salmonellosis
					Chlamydophilosis
					Tuberculosis

On another point of view, pathogen shedding by wild passerine birds could be responsible of maintaining infection in domestic bird pools, such as open-air aviaries or poultry breeding, and could have important economic impact. The presence of *Salmonella* species in starling feces and in cattle feeding operations reported e.g. by Carlson et al. is a good example of an under-known reservoir phenomenon [6]. Another example is the role of birds, including passerines, as amplifying hosts for some vector-borne zoonotic emerging viruses. Open air aviaries are not protected from mosquitoes, and ornamental birds have been shown to be able to act in the same way as their wild counterparts. Migrating birds are also a sanitary concern, since these birds can spread a high variety of pathogens by solely defecating above outdoor aviaries wherein pet birds are housed. Thus this bird concentration could become a non-negligible reservoir of pathogens, contributing to maintain and spread infection in the human population. Referring to vectors, *D. gallinae* according to the author’s advice, is an underestimated concern – probably too many times misdiagnosed - in pet bird medicine as well as in small avian breeding, since the parasite could be carried and transferred from one species to another, mostly by inert materials such as cages, perches, water or feed bowls, etc.… and eventually by man. Threatening pathogens like *C. psittaci* or *Salmonella ssp.* were reported to be carried by the mite and transmitted to pet birds, which could then infect either their owners or their cage mates. In addition, the sanitary state of pet bird owning and trade is rather unclear in many countries. HACCP or other quality control plans (ISO, AFNOR…) are applied by the Federal Agency for Food Safety Chain (FAFSC) in Belgian poultry breeding, but not in “backyard poultry flocks” or in local passerine breeding. Legislation does exist e.g. on international trade but despite this, illegal introduction of birds in our countries still remains a threat for human health when considering the highly pathogenic agents that could be brought in through our frontiers (avian influenza A virus H5N1, chlamydophilosis…).

Therefore, investigating the health status of pet birds, facilities, avian exploitations and owners should be an interesting starting point to define human health risks encountered (from family to breeding scale), to propose economic and sanitary prevention measures (e.g. biosecurity, prophylaxis, hygiene) in an interest of health protection and economic improvement. This investigation could be a good picture illustrating the concept of “Animals + Humans = One Health”.

## 6. Competing interests

The authors declare that they have no competing interests.

## 7. Authors’ contributions

GB and CS fixed the design of the study; GB realized the literature research and analysis; BL, ET, JM and CS were involved in critically revising the manuscript for important intellectual content; CS gave final approval of the version to be published. All authors read and approved the final manuscript.

## 8. Authors’ information

GB is a doctor in veterinary medicine and defended a PhD on songbird behavior and health status. She is currently studying zoonoses transmitted by birds, especially pet birds, in a CS research unit. BL, ET, JGM and CS are professors and heads respectively of parasitology, virology, bacteriology and epidemiology and risk analysis sections of the department of infectious and parasitic diseases, (Faculty of Veterinary Medicine, University of Liège, Belgium) and therefore provided the author with expert advice on diseases discussed in this manuscript.

## References

[B1] Tully TN Jr Elsevier Birds Manual of exotic pet practice 2009 St. Louis, Missouri (USA): Elsevier 250 298

[B2] Dorrestein Gerry M Thomas NTJr, Ecams G, Alan KJ 8 - Passerines Handbook of avian medicine 2009 Second Edinburgh: W.B. Saunders 169 208

[B3] Harcourt–Brown Nigel H Thomas NTJ, Martin PCL, Dorrestein GM Chapter 6 - psittacine birds Avian medicine. edn 2000 Oxford: Butterworth-Heinemann 112 143

[B4] U.S Pet ownership & demographics sourcebook (2007 edition) 2007 Schaumburg, Illinois (USA): American Veterinary Medical Association [https://www.avma.org/KB/Resources/Statistics/Pages/Market-research-statistics-US-Pet-Ownership-Demographics-Sourcebook.aspx]

[B5] Enquête FACCO/TNS SOFRES 2010 sur le parc des animaux familiers français Chambre syndicale des fabricants d’aliments préparés pour chien, chats, oiseaux et autres animaux familiers 2010 Paris (France) [http://www.facco.fr/-Population-animale-]

[B6] Carlson JC Engeman RM Hyatt DR Gilliland RL DeLiberto TJ Clark L Bodenchuk MJ Linz GM Efficacy of European starling control to reduce Salmonella enterica contamination in a concentrated animal feeding operation in the Texas panhandle BMC Vet Res 2011 7 9 10.1186/1746-6148-7-9 21324202 PMC3050709

[B7] Belchior E Barataud D Ollivier R Capek I Laroucau K De Barbeyrac B Hubert B Psittacosis outbreak after participation in a bird fair, Western France, December 2008 Epidemiol Infect 2011 139 1637 1641 10.1017/S0950268811000409 21396150

[B8] Vanrompay D Harkinezhad T Van De Walle M Beeckman D Van Droogenbroeck C Verminnen K Leten R Martel A Cauwerts K Chlamydophila psittaci transmission from pet birds to humans Emerg Infect Dis 2007 13 1108 1110 10.3201/eid1307.070074 18214194 PMC2878242

[B9] Lindeborg M Barboutis C Ehrenborg C Fransson T Jaenson TG Lindgren PE Lundkvist A Nyström F Salaneck E Waldenström J Olsen B Migratory birds, ticks, and crimean-congo hemorrhagic fever virus Emerg Infect Dis 2012 18 2095 2097 10.3201/eid1812.120718 23171591 PMC3557898

[B10] Loye J Carroll S Birds, bugs and blood: avian parasitism and conservation Trends Ecol Evol 1995 10 232 235 10.1016/S0169-5347(00)89072-2 21237019

[B11] Boseret G Carere C Ball GF Balthazart J Social context affects testosterone-induced singing and the volume of song control nuclei in male canaries (Serinus canaria) J Neurobiol 2006 66 1044 1060 10.1002/neu.20268 16838373

[B12] Amonsin A Choatrakol C Lapkuntod J Tantilertcharoen R Thanawongnuwech R Suradhat S Suwannakarn K Theamboonlers A Poovorawan Y Influenza virus (H5N1) in live bird markets and food markets, Thailand Emerg Infect Dis 2008 14 1739 1742 10.3201/eid1411.080683 18976558 PMC2630752

[B13] Wang M Di B Zhou DH Zheng BJ Jing H Lin YP Liu YF Wu XW Qin PZ Wang YL Jian LY Li XZ Xu JX Lu EJ Li TG Xu J Food markets with live birds as source of avian influenza Emerg Infect Dis 2006 12 1773 1775 10.3201/eid1211.060675 17283635 PMC3372357

[B14] Berk Y Klaassen CHW Mouton JW Meis JFGM An outbreak of psittacosis in a bird-fanciers fair in the Netherlands Neth J Med 2008 152 1889 1892 18788682

[B15] Chomel BB Belotto A Meslin FX Wildlife, exotic pets, and emerging zoonoses Emerg Infect Dis 2007 13 6 11 10.3201/eid1301.060480 17370509 PMC2725831

[B16] Van Borm S Thomas I Hanquet G Lambrecht B Boschmans M Dupont G Decaestecker M Snacken R Van Den Berg T Highly pathogenic H5N1 influenza virus in smuggled Thai eagles, Belgium Emerg Infect Dis 2005 11 702 705 10.3201/eid1105.050211 15890123 PMC3320388

[B17] De Schrijver K A psittacosis outbreak in customs officers in Antwerp (Belgium) Bull Inst Marit Trop Med Gdynia 1998 49 97 99 10431651

[B18] Roy L Buronfosse T Using mitochondrial and nuclear sequence data for disentangling population structure in complex pest species: a case study with *dermanyssus gallinae* PLoS One 2011 6 e2230 10.1371/journal.pone.0022305 PMC3143145 21799818

[B19] Karesh WB Cook RA Bennett EL Newcomb J Wildlife trade and global disease emergence Emerg Infect Dis 2005 11 1000 1002 16022772 10.3201/eid1107.050194 PMC3371803

[B20] Karesh WB Cook RA Gilbert M Newcomb J Implications of wildlife trade on the movement of avian influenza and other infectious diseases J Wildl Dis 2007 43 Suppl 3 S55 S59

[B21] Fèvre EM Bronsvoort BMDC Hamilton KA Cleaveland S Animal movements and the spread of infectious diseases Trends Microbiol 2006 14 125 131 10.1016/j.tim.2006.01.004 16460942 PMC7119069

[B22] Brooks-Moizer F Roberton SI Edmunds K Bell D Avian influenza H5N1 and the wild bird trade in Hanoi, Vietnam Ecol Soc 2009 14 1 20

[B23] Valiente Moro C Thioulouse J Chauve C Normand P Zenner L Bacterial taxa associated with the hematophagous mite Dermanyssus gallinae detected by 16S rRNA PCR amplification and TTGE fingerprinting Res Microbiol 2009 160 63 70 10.1016/j.resmic.2008.10.006 19027065

[B24] Valiente Moro C Chauve C Zenner L Vectorial role of some dermanyssoid mites (Acari, Mesostigmata, Dermanyssoidea) Parasite 2005 12 99 109 10.1051/parasite/2005122099 15991823

[B25] Valiente Moro C De Luna CJ Tod A Guy JH Sparagano OAE Zenner L The poultry red mite (Dermanyssus gallinae): a potential vector of pathogenic agents Exp Appl Acarol 2009 48 93 104 10.1007/s10493-009-9248-0 19205905

[B26] Circella E Pugliese N Todisco G Cafiero MA Sparagano OAE Camarda A Chlamydia psittaci infection in canaries heavily infested by Dermanyssus gallinae Exp Appl Acarol 2011 55 329 338 10.1007/s10493-011-9478-9 21761223

[B27] Valiente Moro C Chauve C Zenner L Experimental infection of Salmonella Enteritidis by the poultry red mite, Dermanyssus gallinae Vet Parasitol 2007 146 329 336 10.1016/j.vetpar.2007.02.024 17382475

[B28] Valiente Moro C Desloire S Chauve C Zenner L Detection of Salmonella sp. in Dermanyssus gallinae using an FTA® filter-based polymerase chain reaction Med Vet Entomol 2007 21 148 152 10.1111/j.1365-2915.2007.00684.x 17550434

[B29] Valiente Moro C Desloire S Vernozy-Rozand C Chauve C Zenner L Comparison of the VIDAS® system, FTA® filter-based PCR and culture on SM ID for detecting Salmonella in Dermanyssus gallinae Lett Appl Microbiol 2007 44 431 436 10.1111/j.1472-765X.2007.02119.x 17397483

[B30] Chirico J Eriksson H Fossum O Jansson D The poultry red mite, Dermanyssus gallinae, a potential vector of Erysipelothrix rhusiopathiae causing erysipelas in hens Med Vet Entomol 2003 17 232 234 10.1046/j.1365-2915.2003.00428.x 12823843

[B31] Brännström S Hansson I Chirico J Experimental study on possible transmission of the bacterium Erysipelothrix rhusiopathiae to chickens by the poultry red mite, Dermanyssus gallinae Exp Appl Acarol 2010 50 299 307 10.1007/s10493-009-9317-4 19777357

[B32] Akdemir C Gülcan E Tanritanir P Case report: dermanyssus gallinae in a patient with pruritus and skin lesions Act parasitol 2009 33 242 244 19851974

[B33] Cafiero MA Camarda A Circella E Galante D Lomuto M An urban outbreak of red mite dermatitis in Italy Int J Dermatol 2009 48 1119 1121 10.1111/j.1365-4632.2009.04107.x 19775408

[B34] Cafiero MA Galante D Camarda A Giangaspero A Sparagano O Why dermanyssosis should be listed as an occupational hazard Occup Environ Med 2011 68 628 10.1136/oemed-2011-100002 21486993

[B35] Bellanger AP Bories C Foulet F Bretagne S Botterel F Nosocomial dermatitis caused by Dermanyssus gallinae Infect Control Hosp Epidemiol 2008 29 282 283 10.1086/528815 18205530

[B36] Cafiero MA Camarda A Circella E Santagada G Schino G Lomuto M Pseudoscabies caused by Dermanyssus gallinae in Italian city dwellers: a new setting for an old dermatitis J Eur Acad Dermatol 2008 22 1382 1383 10.1111/j.1468-3083.2008.02645.x 18384564

[B37] Dogramaci AC Culha G Özçelik S Dermanyssus gallinae infestation: an unusual cause of scalp pruritus treated with permethrin shampoo J Dermatol Treat 2010 21 319 321 10.3109/09546630903287437 20687864

[B38] Haag-Wackernagel D Bircher AJ Ectoparasites from feral pigeons affecting humans Dermatology 2010 220 82 92 10.1159/000266039 20016127

[B39] Roy L Dowling APG Chauve CM Lesna I Sabelis MW Buronfosse T Molecular phylogenetic assessment of host range in five Dermanyssus species Exp Appl Acarol 2009 48 115 142 10.1007/s10493-008-9231-1 19160062

[B40] Roy L Ecologie évolutive d’un genre d’acarien hématophage: approche phylogénétique des délimitations interspécifiques et caractérisation comparative des populations de cinq espèces du genre Dermanyssus (Acari: Mesostigmata). articles 2009 Lyon, France: Ecole nationale Vétérinaire de Lyon

[B41] Dorrestein GM Bacterial and parasitic diseases of passerines Vet Clin North Am Exot Anim Pract 2009 12 433 451 10.1016/j.cvex.2009.07.005 19732703

[B42] Clayton DH Tompkins DM Ectoparasite virulence is linked to mode of transmission Proc Biol Sci 1994 256 211 217 10.1098/rspb.1994.0072 8058799

[B43] Valera F Casas-Crivillé A Hoi H Interspecific parasite exchange in a mixed colony of birds J Parasitol 2003 89 245 250 10.1645/0022-3395(2003)089[0245:IPEIAM]2.0.CO;2 12760636

[B44] Haag-Wackernagel D Geigenfeind I Protecting buildings against feral pigeons Eur J Wildl Res 2008 54 715 721 10.1007/s10344-008-0201-z

[B45] Roy L Chauve CM Buronfosse T Contrasted ecological repartition of the northern fowl mite ornithonyssus sylviarum (mesostigmata: Macronyssidae) and the chicken red mite dermanyssus gallinae (mesostigmata: Dermanyssidae) Acarologia 2010 50 207 219 10.1051/acarologia/20101958

[B46] Orton DI Warren LJ Wilkinson JD Avian mite dermatitis Clin Exp Dermatol 2000 25 129 131 10733637 10.1046/j.1365-2230.2000.00594.x

[B47] Blitvich BJ Transmission dynamics and changing epidemiology of West Nile virus Anim Health Res Rev 2008 9 71 86 10.1017/S1466252307001430 18348742

[B48] Nemeth N Young G Ndaluka C Bielefeldt-Ohmann H Komar N Bowen R Persistent West Nile virus infection in the house sparrow (Passer domesticus) Arch Virol 2009 154 783 789 10.1007/s00705-009-0369-x 19347246

[B49] Vázquez A Jiménez-Clavero MA Franco L Donoso-Mantke O Sambri V Niedrig M Zeller H Tenorio A Usutu virus: potential risk of human disease in Europe Euro Surveill 2011 16 31:pii=19935. Available online: http://www.eurosurveillance.org/ViewArticle.aspx?ArticleId=19935 21871214

[B50] Turell MJ O’Guinn M Oliver J Potential for New York mosquitoes to transmit West Nile virus Amer J Trop Med Hyg 2000 62 413 414 11037788 10.4269/ajtmh.2000.62.413

[B51] Sardelis MR Turell MJ O’Guinn ML Andre RG Roberts DR Vector competence of three North American strains of Aedes albopictus for West Nile virus J Am Mosq Control Assoc 2002 18 284 289 12542184

[B52] Komar N Langevin S Hinten S Nemeth N Edwards E Hettler D Davis B Bowen R Bunning M Experimental infection of North American birds with the New York 1999 strain of West Nile virus Emerg Infect Dis 2003 9 311 322 10.3201/eid0903.020628 12643825 PMC2958552

[B53] Pfeffer M Dobler G Emergence of zoonotic arboviruses by animal trade and migration Parasit Vectors 2010 3 35 10.1186/1756-3305-3-35 20377873 PMC2868497

[B54] Comstedt P Bergström S Olsen B Garpmo U Marjavaara L Mejlon H Barbour AG Bunikis J Migratory passerine birds as reservoirs of Lyme borreliosis in Europe Emerg Infect Dis 2006 12 1087 1095 10.3201/eid1207.060127 16836825 PMC3291064

[B55] Mathers A Smith RP Cahill B Lubelczyk C Elias SP Lacombe E Morris SR Vary CP Parent CE Rand PW Strain diversity of Borrelia burgdorferi in ticks dispersed in North America by migratory birds J Vector Ecol 2011 36 24 29 10.1111/j.1948-7134.2011.00137.x 21635638

[B56] Evans EE Zoonotic diseases of common pet birds: psittacine, passerine, and columbiform species Vet Clin North Am Exot Anim Pract 2011 14 457 476 10.1016/j.cvex.2011.05.001 21872782

[B57] Van Droogenbroeck C Beeckman DSA Verminnen K Marien M Nauwynck H Boesinghe LT Vanrompay D Simultaneous zoonotic transmission of Chlamydophila psittaci genotypes D, F and E/B to a veterinary scientist Vet Microbiol 2009 135 78 81 10.1016/j.vetmic.2008.09.047 18963600

[B58] Henrion E Trippaerts M Lepage P Severe multiorgan psittacosis in a 10-year-old boy Arch Pediatr 2002 9 810 813 (in French) 10.1016/S0929-693X(01)00993-9 12205791

[B59] (CDC) CfDCap Compendium of measures to control chlamydia psittaci infection among humans (psittacosis) and pet birds (avian chlamydiosis), 1998 MMWR 1998 47 No.RR-10 1 15 9671426

[B60] Hoelzer K Moreno Switt AI Wiedmann M Animal contact as a source of human non-typhoidal salmonellosis Vet Res 2011 42 34 10.1186/1297-9716-42-34 21324103 PMC3052180

[B61] Panigrahy B Clark FD Hall CF Mycobacteriosis in psittacine birds Avian Dis 1983 27 1166 1168 10.2307/1590218 6651704

[B62] Ward MP Ramer JC Proudfoot J Garner MM Juan-Sallés C Wu CC Outbreak of salmonellosis in a zoologic collection of lorikeets and lories (Trichoglossus, Lorius, and Eos spp.) Avian Dis 2003 47 493 498 10.1637/0005-2086(2003)047[0493:OOSIAZ]2.0.CO;2 12887213

[B63] Madewell BR McChesney AE Salmonellosis in a human infant, a cat, and two parakeets in the same household J Am Vet Med Assoc 1975 167 1089 1090 1104547

[B64] Joseph V Infectious and parasitic diseases of captive passerines Semin Avian Exot Pet 2003 12 21 28 10.1053/saep.2003.127878

[B65] Harris JM Zoonotic diseases of birds Vet Clin North Am Small Anim Pract 1991 21 1289 1298 1767475 10.1016/s0195-5616(91)50139-x

[B66] Grimes JE Zoonoses acquired from pet birds Vet Clin North Am Small Anim Pract 1987 17 209 218 3551306 10.1016/s0195-5616(87)50613-1

[B67] Smith KE Anderson F Medus C Leano F Adams J Outbreaks of Salmonellosis at elementary schools associated with dissection of owl pellets Vector Zoon Dis 2005 5 133 136 10.1089/vbz.2005.5.133 16011429

[B68] Morishita TY Aye PP Ley EC Harr BS Survey of pathogens and blood parasites in free-living passerines Avian Dis 1999 43 549 552 10.2307/1592655 10494426

[B69] Refsum T Vikøren T Handeland K Kapperud G Holstad G Epidemiologic and pathologic aspects of Salmonella Typhimurium infection in passerine birds in Norway J Wildl Dis 2003 39 64 72 12685069 10.7589/0090-3558-39.1.64

[B70] Wells SJ Fedorka-Cray PJ Dargatz DA Ferris K Green A Fecal shedding of Salmonella spp. by dairy cows on farm and at cull cow markets J Food Prot 2001 64 3 11 11198437 10.4315/0362-028x-64.1.3

[B71] Lahuerta A Westrell T Takkinen J Boelaert F Rizzi V Helwigh B Borck B Korsgaard H Ammon A Mäkelä P Zoonoses in the European Union: origin, distribution and dynamics - the EFSA-ECDC summary report 2009 Euro Surveill 2011 16 13:pii=19832. Available online: http://www.eurosurveillance.org/ViewArticle.aspx?ArticleId=19832 21489375

[B72] Hoop RK Ehrsam H Ossent P Salfinger M Mycobacteriosis of ornamental birds–frequency, pathologo-anatomic, histologic and microbiologic data Berl Munch Tierarztl 1994 107 275 281 (in German) 7945185

[B73] Washko RM Hoefer H Kiehn TE Armstrong D Dorsinville G Frieden TR Mycobacterium tuberculosis infection in a green-winged macaw (Ara chloroptera): report with public health implications J Clin Microbiol 1998 36 1101 1102 9542945 10.1128/jcm.36.4.1101-1102.1998 PMC104697

[B74] Steinmetz HW Rutz C Hoop RK Grest P Bley CR Hatt JM Possible human-avian transmission of Mycobacterium tuberculosis in a green-winged macaw (Ara chloroptera) Avian Dis 2006 50 641 645 10.1637/7609-041906R.1 17274308

[B75] Hoop RK Mycobacterium tuberculosis infection in a canary (Serinus canaria L.) and a blue-fronted Amazon parrot (Amazona amazona aestiva) Avian Dis 2002 46 502 504 10.1637/0005-2086(2002)046[0502:MTIIAC]2.0.CO;2 12061666

[B76] Ledwón A Szeleszczuk P Zwolska Z Augustynowicz-Kopeć E Sapierzyński R Kozak M Experimental infection of budgerigars (Melopsittacus undulatus) with five Mycobacterium species Avian Pathol 2008 37 59 64 10.1080/03079450701802255 18202951

[B77] Grange JM Mycobacterium bovis infection in human beings Tuberculosis 2001 81 71 77 10.1054/tube.2000.0263 11463226

[B78] O’Reilly LM Daborn CJ The epidemiology of mycobacterium bovis infections in animals and man: a review Tubercle Lung Dis 1995 76 Suppl 1 1 46 10.1016/0962-8479(95)90591-x 7579326

[B79] Shitaye EJ Grymova V Grym M Halouzka R Horvathova A Moravkova M Beran V Svobodova J Dvorska-Bartosova L Pavlik I Mycobacterium avium subsp. hominissuis infection in a pet parrot Emerg Infect Dis 2009 15 617 619 10.3201/eid1504.081003 19331752 PMC2671411

[B80] Shitaye JE Halouzka R Svobodova J Grymova V Grym M Skoric M Fictum P Beran V Slany M Pavlik I First isolation of mycobacterium genavense in a blue headed parrot (Pionus menstruus) imported from surinam (South America) to the Czech Republic: a case report Vet Med Czech 2010 55 339 347

[B81] Skoric M Fictum P Frgelecova L Kriz P Slana I Kaevska M Pavlik I Avian tuberculosis in a captured ruppell’s griffon vulture (Gyps ruppellii): a case report Vet Med Czech 2010 55 348 352

[B82] Dahlhausen B Tovar DS Saggese MD Diagnosis of mycobacterial infections in the exotic pet patient with emphasis on birds Vet Clin North Am Exot Anim Pract 2012 15 71 83 10.1016/j.cvex.2011.11.003 22244114

[B83] Yogasundram K Shane SM Harrington KS Prevalence of Campylobacter jejuni in selected domestic and wild birds in Louisiana Avian Dis 1989 33 664 667 10.2307/1591142 2619661

[B84] Wedderkopp A Madsen AM Jørgensen PH Incidence of Campylobacter species in hobby birds Vet Rec 2003 152 179 180 10.1136/vr.152.6.179 12622291

[B85] Sensale M Cuomo A Dipineto L Santaniello A Calabria M Menna LF Fioretti A Survey of Campylobacter jejuni and Campylobacter coli in different taxa and ecological guilds of migratory birds Ital J An Sci 2006 5 291 294

[B86] Adhikari B Connolly JH Madie P Davies PR Prevalence and clonal diversity of Campylobacter jejuni from dairy farms and urban sources N Z Vet J 2004 52 378 383 10.1080/00480169.2004.36455 15768139

[B87] Colles FM Dingle KE Cody AJ Maiden MCJ Comparison of Campylobacter populations in wild geese with those in starlings and free-range poultry on the same farm Appl Environ Microbiol 2008 74 3583 3590 10.1128/AEM.02491-07 18390684 PMC2423018

[B88] Waldenström J Axelsson-Olsson D Olsen B Hasselquist D Griekspoor P Jansson L Teneberg S Svensson L Ellström P Campylobacter jejuni colonization in wild birds: results from an infection experiment PLoS One 2010 5 e9082 10.1371/journal.pone.0009082 20140204 PMC2816703

[B89] Olsen B Gylfe A Bergström S Canary finches (Serinus canaria) as an avian infection model for Lyme borreliosis Microb Pathog 1996 20 319 324 10.1006/mpat.1996.0030 8831827

[B90] Rosskopf WJ Jr Common conditions and syndromes of canaries, finches, lories and lorikeets, lovebirds, and macaws Semin Avian Exot Pet 2003 12 131 143 10.1053/seap.2003.00023-9

[B91] Jorn KS Thompson KM Larson JM Blair JE Polly can make you sick: pet bird-associated diseases Clev Clin J Med 2009 76 235 243 10.3949/ccjm.76a.08018 19339639

[B92] Kauffman MD LeJeune J European starlings (sturnus vulgaris) challenged with escherichia coli O157 can carry and transmit the human pathogen to cattle Lett Appl Microbiol 2011 53 596 601 10.1111/j.1472-765X.2011.03163.x 21985308

[B93] Williams ML Pearl DL LeJeune JT Multiple-locus variable-nucleotide tandem repeat subtype analysis implicates European starlings as biological vectors for Escherichia coli O157:H7 in Ohio, USA J Appl Microbiol 2011 111 982 988 10.1111/j.1365-2672.2011.05102.x 21762472

[B94] Gaukler SM Linz GM Sherwood JS Dyer NW Bleier WJ Wannemuehler YM Nolan LK Logue CM Escherichia coli, salmonella, and mycobacterium avium subsp. paratuberculosis in Wild European starlings at a Kansas cattle feedlot Avian Dis 2009 53 544 551 10.1637/8920-050809-Reg.1 20095155

[B95] Perkins LEL Swayne DE Varied pathogenicity of a Hong Kong-origin H5N1 avian influenza virus in four passerine species and budgerigars Vet Pathol 2003 40 14 24 10.1354/vp.40-1-14 12627709

[B96] Fereidouni SR Ziegler U Linke S Niedrig M Modirrousta H Hoffmann B Groschup MH West Nile virus monitoring in migrating and resident water birds in Iran: are common coots the main reservoirs of the virus in wetlands? Vector Borne Zoonotic Dis 2011 11 1377 1381 10.1089/vbz.2010.0244 21923253

[B97] Rutz C Dalessi S Baumer A Kestenholz M Engels M Hoop R Avian influenza: wildbird monitoring in Switzerland between 2003–2006 Schweiz Arch Tierheilkd 2007 149 501 509 (in German) 10.1024/0036-7281.149.11.501 18085164

[B98] Carboni DA Nevarez JG Tully TN Jr Evans DE West Nile virus infection in a sun conure (Aratinga solstitialis) J Avian Med Surg 2008 22 240 245 10.1647/2005-016.1 19014098

[B99] Chvala S Bakonyi T Bukovsky C Meister T Brugger K Rubel F Nowotny N Weissenböck H Monitoring of Usutu virus activity and spread by using dead bird surveillance in Austria, 2003–2005 Vet Microbiol 2007 122 237 245 10.1016/j.vetmic.2007.01.029 17346908

[B100] Chvala S Kolodziejek J Nowotny N Weissenböck H Pathology and viral distribution in fatal Usutu virus infections of birds from the 2001 and 2002 outbreaks in Austria J Comp Pathol 2004 131 176 185 10.1016/j.jcpa.2004.03.004 15276857

[B101] Doneley RJT Miller RI Fanning TE Proventricular dilatation disease: an emerging exotic disease of parrots in Australia Aust Vet J 2007 85 119 123 10.1111/j.1751-0813.2007.00109.x 17359314 PMC7159114

[B102] Honkavuori KS Shivaprasad HL Williams BL Quan PL Hornig M Street C Palacios G Hutchison SK Franca M Egholm M Briese T Lipkin WI Novel Borna virus in psittacine birds with proventricular dilatation disease Emerg Infect Dis 2008 14 1883 1886 10.3201/eid1412.080984 19046511 PMC2634650

[B103] Staeheli P Sauder C Hausmann J Ehrensperger F Schwemmle M Epidemiology of Borna disease virus J Gen Virol 2000 81 2123 2135 10950968 10.1099/0022-1317-81-9-2123

[B104] Pearson GL McCann MK The role of indigenous wild, semidomestic, and exotic birds in the epizootiology of velogenic viscerotropic Newcastle disease in Southern California, 1972–1973 J Am Vet Med Assoc 1975 167 610 614 1176357

[B105] Barin A Arabkhazaeli F Rahbari S Madani SA The housefly, Musca domestica, as a possible mechanical vector of Newcastle disease virus in the laboratory and field Med Vet Entomol 2010 24 88 90 10.1111/j.1365-2915.2009.00859.x 20377736 PMC7168502

[B106] Dubey JP A review of toxoplasmosis in wild birds Vet Parasitol 2002 106 121 153 10.1016/S0304-4017(02)00034-1 12031816

[B107] Dubey JP Hamir AN Experimental toxoplasmosis in budgerigars (Melopsittacus undulatus) J Parasitol 2002 88 514 519 12099420 10.1645/0022-3395(2002)088[0514:ETIBMU]2.0.CO;2

[B108] Wu Y Du PC Li WG Lu JX Identification and molecular analysis of pathogenic yeasts in droppings of domestic pigeons in Beijing, China Mycopathologia 2012 174 203 214 10.1007/s11046-012-9536-9 22476559

[B109] Brilhante RSN Castelo-Branco DSCM Soares GDP Astete-Medrano DJ Monteiro AJ Cordeiro RA Sidrim JJC Rocha MFG Characterization of the gastrointestinal yeast microbiota of cockatiels (Nymphicus hollandicus): a potential hazard to human health J Med Microbiol 2010 59 718 723 10.1099/jmm.0.017426-0 20150318

[B110] Lugarini C Goebel CS Condas LAZ Muro MD De Farias MR Ferreira FM Vainstein MH Cryptococcus neoformans isolated from Passerine and Psittacine bird excreta in the state of Paraná, Brazil Mycopathologia 2008 166 61 69 10.1007/s11046-008-9122-3 18459065

[B111] Papini R Girivetto M Marangi M Mancianti F Giangaspero A Endoparasite infections in pet and zoo birds in Italy Sci World J 2012 2012 253127 10.1100/2012/253127 PMC3317575 22536128

[B112] Filippich LJ McDonnell PA Munoz E Upcroft JA Giardia infection in budgerigars Aust Vet J 1998 76 246 249 10.1111/j.1751-0813.1998.tb10148.x 9612542

[B113] Yaoyu F Xiao L Zoonotic potential and molecular epidemiology of Giardia species and giardiasis Clin Microbiol Rev 2011 24 110 140 10.1128/CMR.00033-10 21233509 PMC3021202

[B114] Joachim A Human cryptosporidiosis: an update with special emphasis on the situation in Europe J Vet Med B 2004 51 251 259 10.1111/j.1439-0450.2004.00765.x 15458486

[B115] Farthing MJ Clinical aspects of human cryptosporidiosis Contrib Microbiol 2000 6 50 74 10943507 10.1159/000060368

[B116] Quah JX Ambu S Lim YAL Mahdy MAK Mak JW Molecular identification of Cryptosporidium parvum from avian hosts Parasitology 2011 138 1 5 21232175 10.1017/S0031182010001691

[B117] Cray C Infectious and zoonotic disease testing in pet birds Clin Lab Med 2011 31 71 85 10.1016/j.cll.2010.10.008 21295723

[B118] Bernard PP-PV A global veterinary education to cope with societal needs Proceedings of the OIE global conference on evolving veterinary education for a safer world OIE, 12–14 October 2009 2009 Paris (France): Office International des Epizooties 15 22

